# Effect of Clamping Compression on the Mechanical Performance of a Carbon Paper Gas Diffusion Layer in Polymer Electrolyte Membrane Fuel Cells

**DOI:** 10.3390/membranes12070645

**Published:** 2022-06-23

**Authors:** Yanqin Chen, Jinghui Zhao, Cuihong Jin, Yuchao Ke, Decai Li, Zixi Wang

**Affiliations:** 1 Department of Mechanical Engineering, Tsinghua University, Beijing 100084, China; yanqin.chen@outlook.com (Y.C.); lidecai@mail.tsinghua.edu.cn (D.L.); 2 School of Automotive Engineering, Tongji University, Shanghai 201804, China; zhaojinghui@tongji.edu.cn; 3 New Energy R&D Center, Weichai Power Co., Ltd., Weifang 261061, China; jincuihong@weichai.com; 4 Key Laboratory of High-Performance Rubber & Products of Anhui Province, Ningguo 242300, China; keyc@zhongdinggroup.com

**Keywords:** gas diffusion layer, polymer electrolyte membrane fuel cells, clamping compression, nonlinear characteristics, mechanical failures

## Abstract

During all the assembly stages of a polymer electrolyte membrane fuel cell (PEMFC) stack, gas diffusion layers (GDLs) endure clamping loads in the through-plane direction several times. Under such complicated assembly conditions, GDLs have to deform with the changes in structure, surface roughness, pore size, etc. A comprehensive understanding of the compressive performance of GDLs at different clamping phases is crucial to the assembly process improvement of PEMFCs. Two typical clamping compression was designed and performed to get close to the actual assembly conditions of PEMFCs. The results indicate that the initial clamping compression and the magnitude of the maximum clamping load have great impacts on the segmented compressive properties of GDLs. The nonlinear compressive performance of the GDL is mainly attributed to the unique microstructural information. The rough surface morphology contributes to the initial compressive characteristics where the big strain along with the small stress occurs, and the irreversible failures such as carbon fiber breakages and adhesive failures between fibers and binders account for the hysteresis between different compression stages. Importantly, it is found that the clamping compression hardly influences the small pore distribution below 175 μm but affects the large pore distribution over 200 μm.

## 1. Introduction

Polymer electrolyte membrane fuel cells (PEMFCs), as the most promising candidate of fuel cells [1], has been attracted more and more attention owing to their outstanding performance such as high efficiency, low emissions, quick startup time, etc. In general, a typical PEMFC unit consists of a membrane electrode assembly (MEA) in the middle and the anode and cathode bipolar plates (BPPs). All the components are tightly clamped together by endplates with bolts and nuts [2,3]. An MEA with five-layered structures is traditionally composed of a polymer electrode membrane (PEM), the anode and cathode catalyst layers (CLs), and the anode and cathode gas diffusion layers (GDLs). With the improvement of the industrialization and manufacturing techniques of fuel cells, an MEA has another two novel three-layered structures [4], i.e., the anode and cathode gas diffusion electrodes (GDEs) together with a PEM, as well as the coated catalyst layer (CCL) together with the anode and cathode GDLs. In any case, all the individual elements in an MEA with five-layered or three-layered structures have to be tightly clamped together under a certain pressure by adjusting the compression ratio, torque, plate pressure, and clamping force [5]. A single PEMFC unit normally generates a voltage below 1 V [6], which is far limited for practical use. To meet the requirement and achieve sufficient power, specific numbers of PEMFC units have to be assembled and packaged together under a certain pressure to form a stacked structure. Even though PEMFCs are applied in transportation, stationary, and portable equipment, some challenges [7,8] still exist such as costs, durability, service life, power efficiency, degradation issues, structural failures, etc. On the whole, during all the assembly processes from individual components to an overall PEMFC stack, all the elements have to experience various degrees of clamping loads several times. Excessive clamping compression leads to some structural failures such as internal cracks and breakages of carbon fibers in GDLs [9], fiber intrusion into gas channels, and microporous layer (MPL) penetration into CL, while mild clamping stress results in leakage issues and worse contact conditions [10]. The compressive characteristics of GDLs play a significant role in influencing cell performance such as heat and mass transfer [11], bulk and pore characteristics [12], gas permeability [13], etc. It is very significant and fundamental to investigate how the mechanical performance of GDLs behaves during the different assembly stages of a PEMFC stack.

Carbon paper-type GDLs, generally with a global thickness of 100–400 μm [14], are widely used in PEMFCs. They are typically made of a substrate with carbon fibers and an MPL that is composed of carbon powder and coated with polytetrafluoroethylene (PTFE). With the brittle characteristic of carbon fibers, irreversible breakages and cracks easily occur when GDLs suffer mechanical pressure [15]. Importantly, the performance of GDLs such as porosity [16], gas permeability [17], electrical and thermal conductivity [18], morphology, and wettability [19] varies with the mechanical loads. To summarize, once the applied clamping pressure on GDLs changes, GDLs deform along with the changes in surface morphology, internal network of carbon fibers, contact conditions, etc. It has been experimentally investigated that the compressive behavior of GDLs is nonlinear [20,21]. Researchers have made great efforts to model the compressive nonlinearity of GDLs by polynomial function and piece-wise functions [22]. However, the overdependence on experimental results by curve fitting narrows the application of these compressive nonlinear models. In addition, the prominent achievements of GDLs under different compression conditions are summarized in Table 1.

Although the compressive properties of GDLs have been investigated, the majority of the research focused on how the mechanical performance behaves under cyclic compression with constant loads and simplified clamping conditions. In practice, the clamping stress during all the assembly processes of a PEMFC stack is extremely complicated even with variable compression load magnitudes. Studying the mechanical properties of GDLs under the clamping conditions closed to the actual assembly procedures of a PEMFC stack is significant for the reliable stress simulation of fuel cells, which can build an accurate link between mechanical properties, and the other performance such as porosity, electrical and thermal conductivity, contact resistance, etc. The present study experimentally investigated the mechanical response of a commercial carbon paper GDL to a series of clamping compression. Particularly, two representative clamping conditions with constant maximum loads and variable maximum loads were designed and performed to stimulate the assembly processes of a PEMFC stack. Furthermore, the pristine and compressed GDLs were characterized by thickness, surface morphology, roughness, and pore size distribution. All the achieved structural information was employed to interpret the failure mechanism and reveal the effects of clamping compression on the mechanical performance of GDLs.

## 2. Experimental Details

Carbon paper-based GDLs are commonly composed of a substrate with randomly arranged carbon fibers and an MPL coated with PTFE. In general, the thickness of a substrate almost accounts for over 70% of the total thickness of GDLs. Carbon fibers are anisotropic with a longitudinal modulus of 225 GPa and a transverse modulus of 15 GPa [31], and the PTFE in MPL has a storage modulus of 1 GPa [32]. Compared to the MPL, carbon fibers seem to contribute more to the nonlinear and anisotropic characteristics of GDLs. As a typical carbon paper GDL, the Toray series have been investigated owing to their wide applications in PEMFCs. With only one substrate layer, pristine Toray GDLs could be redesigned and treated with various concentrations of PTFE and different thicknesses of MPL to meet specific needs. In this study, TGP-H-060 GDLs from the Toray series were employed for experiments. Some specifications of the carbon paper GDL provided by the supplier are listed in Table 2.

### 2.1. Clamping Compression Experiments

**Figure 1:**
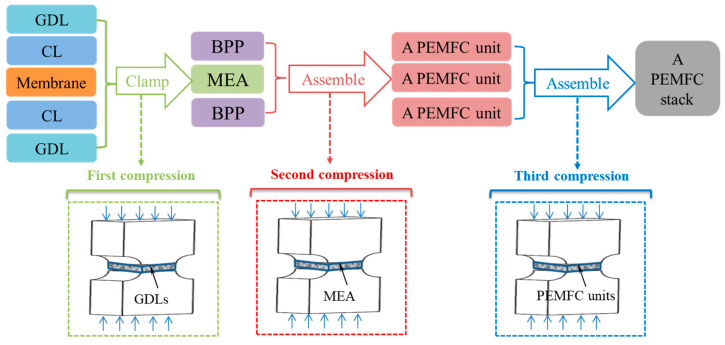
Schematics of the assembly processes of a PEMFC stack.

Normally, conventional assembly processes of a PEMFC stack can be summarized in three key steps, as presented in Figure 1. During the fabrication of an MEA by either hot-press or without-hot-press way, GDLs are initially compressed by pressing plates and then fastened by bolts [5]. In the following, an MEA with anode and cathode BPPs is tightly clamped to compose a PEMFC unit. In the above two assembly processes, clamping loads directly act on GDLs. In practice, a single PEMFC cell just converts chemical energy into electricity with extremely low power. To meet practical needs, multi-layered cells are very necessary to be packaged together to form a large PEMFC stack in order to generate reasonable power. In the third assembly step, the clamping load is mostly applied on endplates and current collectors.

The mechanical performance changes of GDLs caused by clamping loads play an important role in influencing the overall performance of PEMFCs [9]. With regard to clamping conditions and load values during the entire assembly stages of PEMFCs, typical findings are given in Table 3. It can be concluded that the applied way and magnitudes of clamping loads vary a lot during the assembly processes of an MEA, a single fuel cell unit, and a PEMFC stack. In the current, there are no standards or criteria to determine a reasonable magnitude of the clamping loads in each step, brittle characters of carbon paper GDLs make it very sensitive to the maximum compressive pressure [33,34]. It is very valuable to dig out how GDLs behave in such complicated clamping conditions.

**Figure 2:**
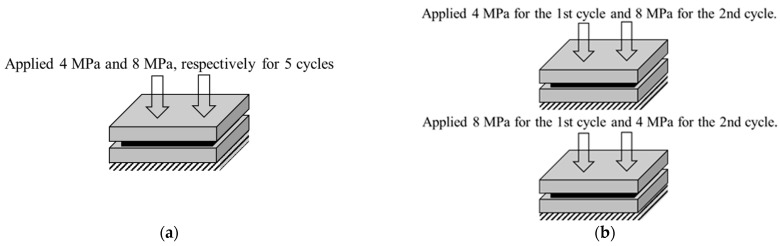
Details of the two representative clamping compression with constant pressure in (**a**) and variable pressure in (**b**).

Due to the uncertainty of clamping loads in each assembly step, this research designed two typical compression conditions to stimulate the clamping processes. In the first case, GDLs were performed cyclic compression for five cycles. In the cyclic compression, the maximum pressure was kept constant, and two maximum loads were employed. In the second case, GDLs were conducted with compression with variable loads twice. Details of the clamping compression are illustrated in Figure 2. All the compression tests were performed by a universal tester (SHIMADZU AG-X, Kyoto, Japan) with a load precision of ±0.5% and resolution of 0.12 μm, under a speed of 0.01 mm/min, at room condition (with a temperature of 20–25 °C). Besides, the applied two maximum compressive loads were selected as 4 MPa and 8 MPa in order to cover the clamping load range as listed in Table 3.

### 2.2. Structural Characterization of the Carbon Paper GDL

**Figure 3:**
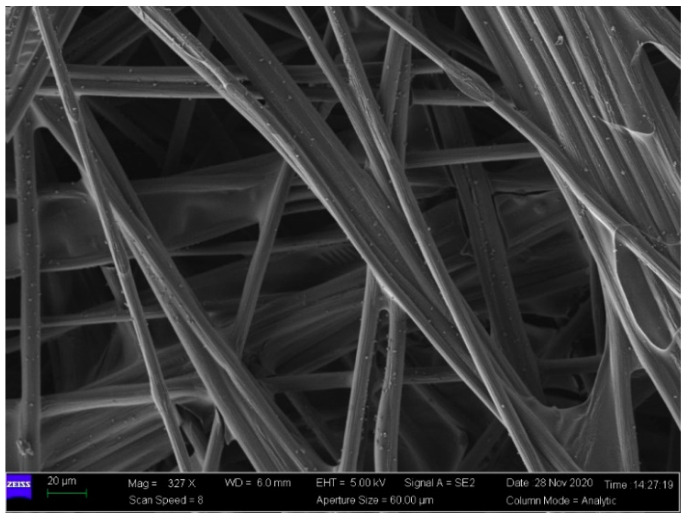
A representative SEM image of a pristine TGP-H-060 GDL.

In addition to clamping compression experiments, the surface morphology of GDLs was observed by a scanning electron microscope (SEM, Zeiss Gemini 300, Oberkochen, Germany). A representative SEM image of a pristine GDL is shown in Figure 3. It can be seen that the network of the fresh GDL is well connected by randomly arranged carbon fibers without any breakages or cracks, and all the fibers are bonded together by adhesive binders as a result of porous structures. The multi-point supporting network of GDLs leads to inhomogeneous and extremely rough contact surfaces. Such heterogeneous surfaces are very sensitive to clamping loads. What is worse, irreversible damages to carbon fibers easily occur with the increase in applied pressure [34].

**Figure 4:**
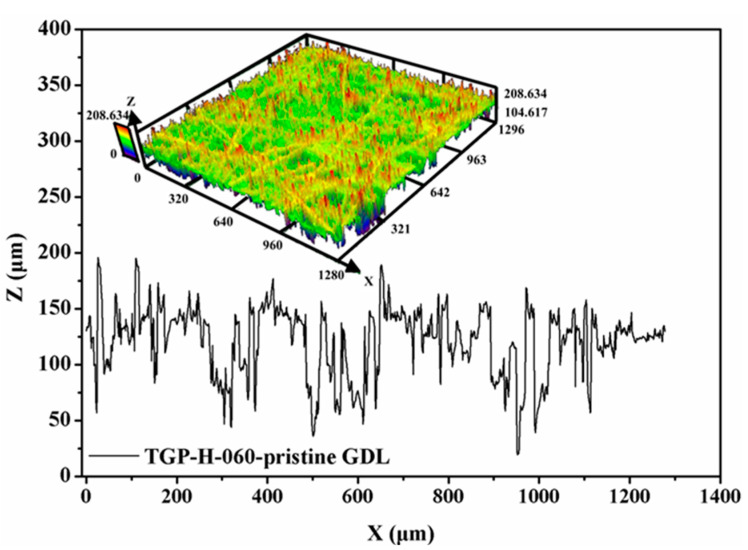
Surface roughness of a pristine TGP-H-060 GDL.

Although the SEM apparatus is good at the microstructure observation in two dimensions (or in-plane directions) in the micro scale size, it has a very limited ability to exhibit the structural characteristics of GDLs in a large scale size, particularly in the through-plane direction. With a confocal laser microscope (LEXT OLS5100, Tokyo, Japan), more morphology information of GDLs can be captured in a larger scale, especially surface roughness. The surface profile and roughness of a pristine GDL are presented in Figure 4. Even the thickness of a fresh GDL with 190 μm is quite thin, its surface morphology is extremely rough. In reality, applied clamping loads on GDLs affect the roughness to some extent, which directly determines the effective contact conditions between GDL and other components [18,25,48].

**Figure 5:**
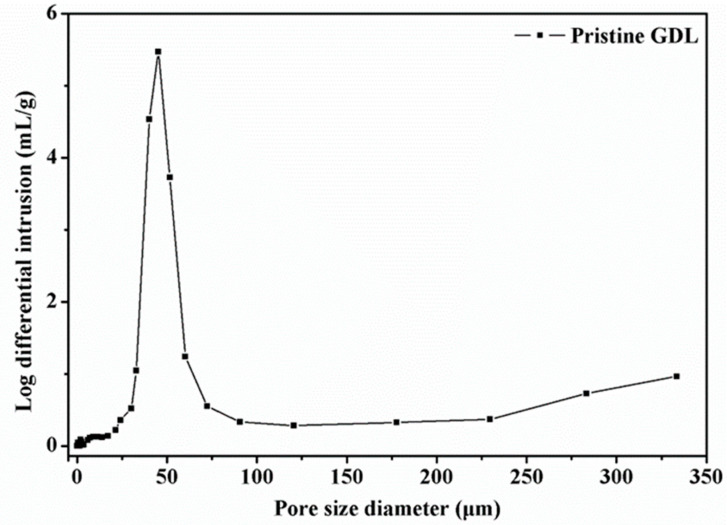
Pore size distribution of a pristine TGP-H-060 GDL.

With SEM and surface roughness images, it can be intuitively observed the appearance profile and architecture of GDLs. To quantitatively characterize the effective structural characteristics of GDLs, an automatic mercury porosimeter (Auto Pore V9620, Atlanta, GA, USA) was employed to measure the pore size distribution. The original pore size distribution of a pristine GDL is described in Figure 5. It can be found that the pore size of the fresh GDL distributes with a wide range from nanometer to micrometer, especially concentrates from 25 μm to 75 μm, with a peak value around 50 μm.

Compared to pristine GDLs, compressed GDLs after a series of clamping compression were also observed with SEM images and surface roughness and measured with the pore size distribution. With these elaborate structural descriptions of GDLs, it could help us further understand the structural changes caused by clamping pressure and guide us to a reasonable assembly procedure of a PEMFC stack with excellent performance.

## 3. Results and Discussion

From individual elements to an overall PEMFC stack, GDLs suffer clamping compression several times due to the specific assembly procedures. Even in a running PEMFC stack, GDLs still service under a certain compressive load. This research mainly focused on investigating the mechanical performance of a carbon paper-type GDL under different clamping conditions. Two typical clamping compression with homogeneous stress was designed and performed. The key findings can be found as follows.

### 3.1. Mechanical Performance of GDLs under Cyclic Compression with Constant Maximum Clamping Loads

**Figure 6:**
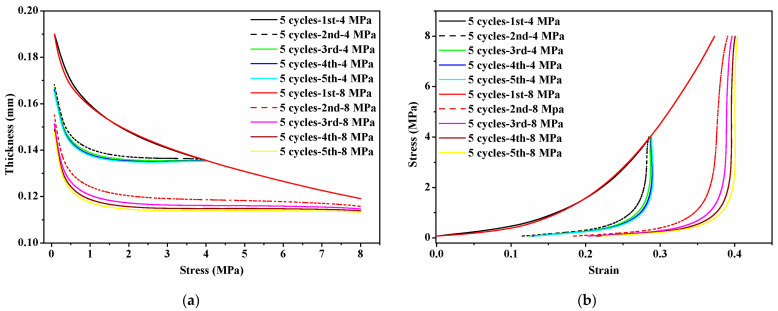
Mechanical performance of GDLs under cyclic compression with two constant clamping loads for five cycles: (**a**) thickness vs. stress and (**b**) stress vs. strain.

Figure 6 shows the mechanical behavior of GDLs under two maximum compressive loads of 4 MPa and 8 MPa for five cycles. In Figure 6a, it can be seen that the thickness of the GDL gradually decreases with the increase in applied stress during the initial compression. However, from the second to the fifth compression, the thickness decreases at the beginning stage of applying clamping load from 0 to 1 MPa and then comes to plateaus. Meanwhile, the thickness of GDLs after two cycles (from the third to the fifth compression) hardly changes. After calculation, the relationship between thickness and stress can be converted into compressive stress vs. strain curves as presented in Figure 6b. The compressive performance of the GDL behaves in a nonlinear manner. During the first compression, the mechanical behavior of the GDL subject to 4 MPa and 8 MPa shares the same tendency. Remarkably, there is a big gap in the compressive stress vs. strain curves between the first compression and the other following four compressions, which shows a good agreement with the findings in [23,49,50]. The GDL is very sensitive to clamping loads, and the initial clamping compression greatly influences its compressive performance. While the continuous compression from the third to the fifth cycle up to the same maximum clamping load weakly affects the compressive properties of GDLs. It can be concluded that the magnitude of the maximum clamping load during the initial compression applied on GDLs plays a decisive role in the determination of the compressive strain range and distribution.

**Figure 7:**
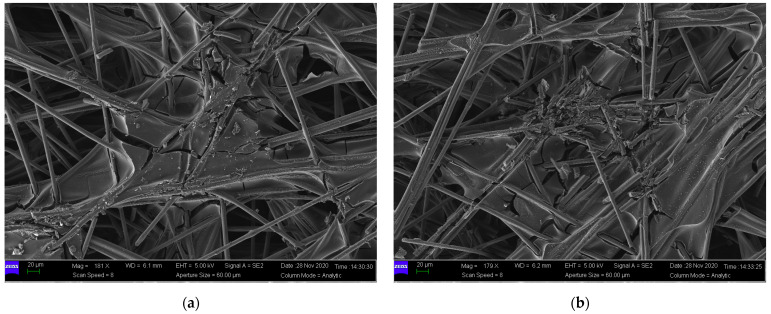
SEM images of compressed GDLs with a maximum load of: (**a**) 4 MPa and (**b**) 8 MPa.

In the current, the mechanical performance of GDLs under cyclic compression has been widely investigated [23,49,50] with nonlinear characteristics. However, most of these contributions did not give convincing explanations of the nonlinearity in detail. The present study made great efforts to interpret the mechanism of the compressive properties of GDLs through their microstructural information. SEM images of compressed GDLs subject to 4 MPa and 8 MPa are given in Figure 7. Compared to that of the pristine GDL in Figure 3, it can be seen that the structures of compressed GDLs after cyclic compression have been destroyed with two typical irreversible failures such as breakages of carbon fibers and the adhesive fractures between binders and fibers. In addition, it can be observed that the mechanical failure degree of GDLs subject to 8 MPa is more serious than that of GDLs subject to 4 MPa to some extent, as shown in Figure 7.

**Figure 8:**
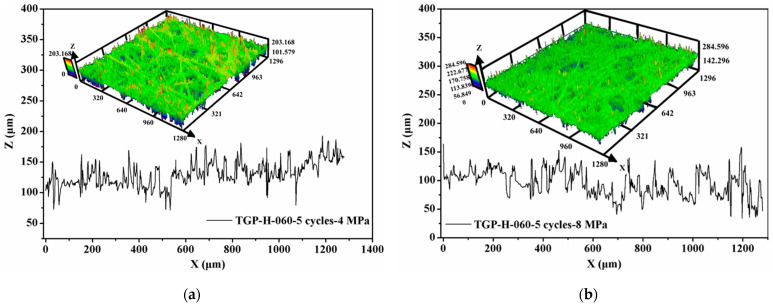
Surface profile of compressed GDLs with a maximum load of: (**a**) 4 MPa and (**b**) 8 MPa.

Besides, the surface profile of compressed GDLs was observed and their surface roughness was also measured, as exhibited in Figure 8. Compared to that of the fresh GDL as given in Figure 4, the surface of compressed GDLs tends to be a little flat. The fluctuations in the Z direction (or through-plane direction) of GDLs in Figure 4 and Figure 8 contribute to the specific mechanical performance at the initial stage where the big strain along with the small stress occurs as shown in Figure 6b. With the increase in clamping loads, the thickness of GDLs becomes solid until to a relatively stable condition with a certain value under different maximum loads as shown in Figure 6a. That indicates GDLs reach a relatively firm state and show a strongly compressive ability to resist the external pressure where the small strain along with the big stress happens, as shown in Figure 6b. In conclusion, the maximum clamping loads applied on GDLs during the initial compression determine their mechanical failure degrees and compressive characteristics.

### 3.2. Mechanical Performance of GDLs under Repetitive Compression with Variable Maximum Clamping Loads

**Figure 9:**
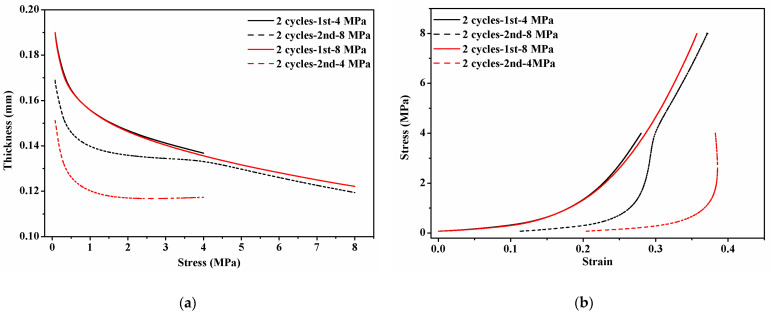
Compressive performance of GDLs under variable clamping loads: (**a**) thickness vs. stress and (**b**) stress vs. strain.

During all the assembly processes of a PEMFC stack, the clamping loads are flexible, and they cannot keep constant in each assembly step. Besides constant maximum clamping loads, it is also very significant to investigate how the mechanical performance of GDLs behaves under variable maximum clamping loads. In the previous section, it can be found that the initial compression plays a crucial role in the determination of the mechanical performance of GDLs, and the continuous compression after two cycles makes no big difference. This section concentrated on the impacts of variable maximum clamping loads on GDLs. Figure 9 exhibits the mechanical characteristics of GDLs under two representative variable clamping loads, such as applying 4 MPa for the first compression and 8 MPa for the second compression, as well as applying 8 MPa for the first compression and 4 MPa for the second compression. Remarkably, the mechanical behavior of GDLs for the second compression as dashed red and black lines marked in Figure 9 behaves in a complicated manner.

The dashed black line in Figure 9a can be divided into three sections. In the first section with the stress from 0 to 1 MPa, the thickness of the GDL rapidly decreases at the very beginning stage of applying pressure. After 1 MPa until 4 MPa, it gradually reaches a plateau, which is similar to that shown in Figure 6a. However, in the last section with the stress from 4 MPa to 8 MPa, the thickness decreases with the same tendency as the red solid line in Figure 9a. A similar phenomenon can be found in the compressive stress vs. strain curves in Figure 9b. The segmented characteristics in the compressive performance of the GDL in the second compression reveal that GDLs are very sensitive to the maximum clamping loads, even though they have been performed during the first clamping compression. As for the dashed red line in Figure 9a, the segmented characteristics do not occur. In a word, after GDLs experience the initial compression with a certain clamping load, in the following continuous compression, their compressive performance almost keeps stable if the applied pressure is below the first clamping load; while their compressive characteristics behave in a novel way with accumulating mechanical deformation if the applied pressure is beyond the first clamping value.

**Figure 10:**
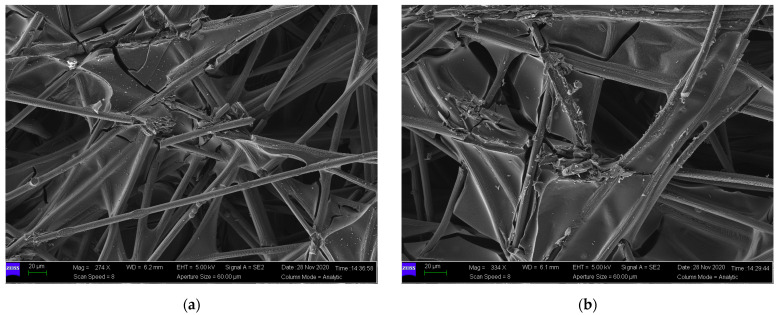
SEM images of GDLs after two compression: (**a**) firstly with 4 MPa and secondly with 8 MPa and (**b**) firstly with 8 MPa and secondly with 4 MPa.

By SEM images as shown in Figure 3, Figure 7 and Figure 10, it can be seen that clamping loads applied on GDLs result in irreversible failures. To some extent, the magnitude of maximum clamping loads positively contributes to irreversible damage degrees, and they could be used to explain the segmented characteristics in the compressive performance of GDLs when they suffer several maximum pressures during all the assembly stages of a PEMFC stack.

**Figure 11:**
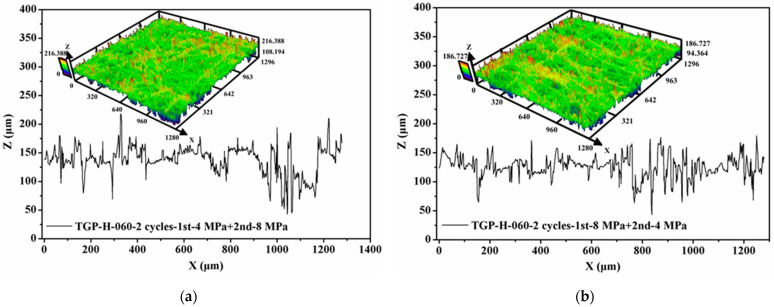
Surface profile of GDLs after two compression: (**a**) firstly with 4 MPa and secondly with 8 MPa and (**b**) firstly with 8 MPa and secondly with 4 MPa.

Besides SEM images, the surface profile of GDLs after repetitive compression with variable maximum clamping loads was observed, and their surface roughness was measured, as exhibited in Figure 11. Even GDLs experienced clamping compression twice, the surface of compressed GDLs turns flat but is still rough, and the surface roughness takes the responsibility for the initial compressive characteristic with large strain along with small stress.

**Figure 12:**
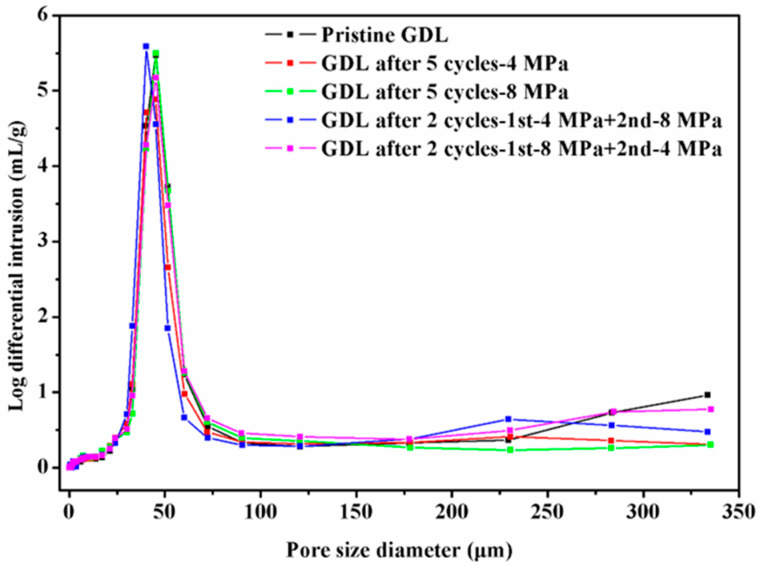
Pore size distribution of pristine and compressed GDLs.

It is not convincing to use the appearance architecture of GDLs to represent the overall structural characteristics of GDLs without any descriptions of their interior structure. In the current, it is almost impossible to observe the internal structure of compressed GDLs without any damage treatment due to the lack of reliable experimental methods. In this study, the pore size distribution of GDLs measured by an automatic mercury porosimeter was employed to quantitatively state the effective structural characteristics, as presented in Figure 12.

In general, the pore size of pristine and compressed GDLs mostly concentrates on a range from 25 μm to 75 μm. Even though the clamping compression barely influences the small pore size distribution where the pore diameter is below 175 μm, it affects the large pore size distribution where the pore size diameter is over 200 μm. The pristine GDL has the most pore size diameter beyond 300 μm, compared to compressed GDLs. The fact that broken fibers and binders fall into pores and fill voids might account for this phenomenon. Notably, even though the magnitude of maximum clamping loads shows great impacts on the compressive performance and mechanical failure degrees of GDLs, it exhibits minor effects on the pore size distribution.

## 4. Conclusions

This research focused on the impacts of clamping loads on the mechanical performance of a commercial carbon paper-type GDL. Owing to the complicated assembly procedures of a PEMFC stack, two representative clamping compression with constant and variable maximum clamping loads were designed and performed to get close to real clamping conditions that GDLs endure in fuel cells. By SEM images, surface profile, interfacial roughness, and pore size distribution of GDLs, their structures were characterized to interpret the compressive mechanism.

In conclusion, the mechanical characteristics of the carbon paper GDL without an MPL behave in a nonlinear manner, and the initial clamping compression has a huge influence on its mechanical performance. Significantly, the compressive performance of GDLs is very sensitive to the magnitude of the maximum clamping load. With SEM images, it can be seen that larger clamping loads result in more serious mechanical failures such as breakages of carbon fibers and adhesive failures between binders and fibers. By surface profile, it can be concluded that the surface roughness contributes a lot to the mechanical characteristics of GDLs (where the phenomenon occurs with the big strain along with small stress) at the very beginning stage of applying pressure. In addition, the pore size distribution of GDLs was measured to quantitatively describe their effective structural changes. Although the clamping compression plays a decisive role in the determination of the mechanical performance of GDLs, it hardly influences the distribution of small pores with a diameter under 175 μm but affects the distribution of large pores with a diameter over 200 μm. The findings in this research could guide the assembly procedures and reliable stress simulation of PEMFCs with better performance.

## Figures and Tables

**Table 1:** The mechanical response of GDLs under different compression conditions.

Type of GDLs	Compression Conditions	Key Findings	Sources
GDM (BC-39)	Three cyclic compression was applied with the maximum load of 1 MPa.	An accurate compressible GDM model is proposed to capture the experimental cyclic response.	[23]
Freudenberg H24CX483	Two cyclic compression: one with the stress of 3 MPa and the other with three levels of stress (1 MPa, 2 MPa, 3 MPa).	A new nonlinear constitutive model considering historical maximum stress and a prediction method for cyclic compression properties of GDL are developed.	[21]
TGP-H-120	Cyclic compression was set up to 1.7 MPa and 3.4 MPa for 5 cycles.	Static cyclic compression causes structural and property changes of GDL.	[24]
Toray GDLs	Cyclic compression was between 0 and 8 MPa according to the transmission line method.	The smallest contact resistance.	[25]
SGL GDLs		The highest contact resistance.	
Felt GDLs		The smallest difference rates between the cycles of compression.	
SGL 24AA, 24BA, and 24 BC	High compressive loads were under dynamic excitation and over a large temperature range.	The dynamic compression modulus increases linearly with temperature until 280 °C then it decreases linearly.	[20]
Carbon paper GDL-A and GDL-B	Apply different levels of mechanical stress on two regions.	Stress-relative density curves were built to predict experimental compressive behavior of GDLs.	[26]
Uncoated GDLs(Toray-H-090)	An initial assembling compression was with 0–1 MPa, followed by 10 cycles of loading and unloading between 1 and 3 MPa.	Uncoated GDLs show the least reduction in thickness and gas permeability after compression.	[19]
Coated GDLs(SGL 24BA, 10BA, 34BC, 35BC		SGL 35BC shows substantially much higher reduction in thickness and gas permeability compared to SGL 34BC.	
SGL 29BC	Compression ratio was set with different levels (0, 8.6, 23.6, and 38.6% of the initial thickness).	The average pore diameter of the fibrous substrate reduces with the compression pressure, whereas that of the microporous layer remained unchanged even at high compression (38.6%).	[11]
Carbon paper GDL	Steady load in the constant conditions (2 MPa, 4 MPa, and 6 MPa) and cyclic load up to 6 MPa for 6 cycles.	The electrical resistance decreases as the load cycles increases.	[27]
Woven carbon cloth		More uniform decline of the resistance is caused by the increasing fiber cracks.	
Felt GDL		Tortuous and thick fibers lead to higher stability in electric resistance.	
Reconstructed GDL	Finite element volume method was used to simulate GDL compression with the ratio 0–30%.	Compression reduces the oxygen diffusivity and intrinsic permeability.	[28]
SGL 25BA	Compression ratio ranges from 0 to 49%.	Compression is mainly related to changes in porosity and geodesic tortuosity.	[29]
Simulated multilayered GDLs	Compression ratio ranges from 0 to 30%.	The pore size distribution, permeability, tortuosity, and electric conductivity are influenced with compression.	[30]

**Table 2:** Specifications of TGP-H-060 GDL.

Properties	Value
Thickness	190 μm
Density	0.44 g/cm^3^
Porosity	78%
PTFE treated	No
MPL	No

**Table 3:** Key findings of clamping loads during assembly procedures of PEMFCs.

Components	Applied Load Conditions	Maximum Load	Sources
MEA	Hot-press with 450 psi at 170 °C for 4 min	450 psi	[35]
	Hot-press with 500 and 1500 psi, at 100 and 160 °C for 2 and 5 min, respectively	1500 psi	[36]
	Hot-press with 5000–15,000 KPa at 160–270 °C for 1–5 min	15 MPa	[37]
	Hot-press with 400 psi at 130 °C for 3 min	400 psi	[38]
	Without hot-press under a torque of2 Nm	2 Nm	
A PEMFC unit	Hot-press with stress varying from 0.068 to 13.8 MPa, at 135 °C for 2 min	13.8 MPa	[39]
	13 Nm per bolt, together with pneumatically pressurized pocket end plate pressure up to 7 bars	13 Nm per bolt and 7 bars	[40]
	Assembly pressure from 1.5 MPa to 5.5 MPa	5.5 MPa	[41]
	Bolt torque from 2 to 11 Nm	11 Nm per bolt	[42]
	Plate pressure up to 6 MPa	6 MPa	[43]
	Clamping force from 0 to 400 kgf	400 kgf	[44]
PEMFC stack	1865 N per bolt, 6 cells and 8 bolts for the stack	1865 N per bolt	[45]
	Clamping pressure from 1.5 MPa to 3.5 MPa	3.5 MPa	[22]
	Clamping force per belt from 5 to 7 KN	7 KN per bolt	[46]
	Clamping force per clamping belt from 10 to 35 KN	35 KN per bolt	[47]

## Data Availability

The data presented in this study are available on request from the corresponding author.
